# Using Plant-Derived Polysaccharides in the Management of Cognitive Disorders: Molecular and Clinical Perspectives

**DOI:** 10.3390/nu18040555

**Published:** 2026-02-07

**Authors:** Yandong Li, Linlin Du, Mengyuan Wang, Xingyu He, Zongsuo Liang, Jingling Liu

**Affiliations:** 1 School of Pharmacy, Shaanxi University of Chinese Medicine, Xianyang 712046, China; 2 College of Sciences, Northwest A&F University, Xianyang 712100, China; 3 School of Life Science and Medicine, Zhejiang Sci-Tech University, Hangzhou 310018, China

**Keywords:** plant-derived polysaccharides (PDPs), cognitive impairment and neurodegeneration, neuroprotection, gut–brain axis and microbiota, protein aggregation & apoptosis, clinical translation & RCTs

## Abstract

Background: Cognitive disorders are a class of neurological diseases characterized by declines in cognitive function, imposing a heavy physical and mental burden on middle-aged and elderly individuals. Since the underlying mechanisms of cognitive disorders are not yet fully understood, and different types of cognitive disorders may involve distinct mechanisms, the development of drugs with multi-target therapeutic and preventive effects is of great significance. Currently approved drugs for Alzheimer’s disease, such as acetylcholinesterase inhibitors and NMDA receptor antagonists, show limited efficacy and poor prognosis, highlighting the urgent need for safe and effective alternative treatments. Methods: Plant-derived polysaccharides (PDPs) possess antioxidant, anti-inflammatory, neuroprotective, and immunomodulatory activities, and polysaccharides derived from traditional Chinese medicine, in particular, show great potential for treating cognitive disorders. In recent years, interventions with plant polysaccharides that modulate the gut microbiota to affect the “gut–brain axis” and improve cognitive function have become an emerging research focus. Clinical evidence has also increasingly supported the beneficial effects of polysaccharides on cognitive disorders. Therefore, this study focuses on plant polysaccharides in the intervention of cognitive disorder-related diseases, including both in vivo and in vitro experiments, and summarizes existing findings according to different mechanisms of action. Results and Conclusions: Relevant clinical reports are incorporated to provide a theoretical basis and strong support for the development of polysaccharide-based interventions and new drug development for cognitive disorders.

## 1. Introduction

Cognitive impairment represents a heterogeneous group of neurological disorders. These disorders are characterized by progressive deterioration in memory, executive function, attention, language, and other cognitive domains. The most prevalent forms include Alzheimer’s disease (AD), vascular dementia (VaD), and mild cognitive impairment (MCI). These conditions not only have a profound negative impact on patients’ quality of life but also impose a considerable socioeconomic burden worldwide [1]. With the rapid acceleration of global population aging, the prevalence of age-related cognitive impairment is projected to rise continuously. By 2050, an estimated 152.8 million individuals are expected to be affected [2]. The associated economic costs are primarily attributable to direct medical expenses, productivity loss, and the demand for long-term care [3]. Thus, cognitive impairment has become a pressing global public health issue. Beyond economic implications, affected individuals frequently encounter multidimensional challenges. These include difficulties in social participation, interpersonal relationships, occupational functioning, and overall health status. Despite extensive research efforts, the complex pathophysiological mechanisms underlying cognitive impairment remain incompletely understood. Moreover, currently available pharmacological therapies provide only modest symptomatic relief during the early stages. They do not halt or reverse the progressive trajectory of the disease.

Current pharmacological interventions for cognitive impairment are mainly focused on symptomatic management. The principal therapeutic agents include acetylcholinesterase inhibitors, such as donepezil, and the *N*-methyl-D-aspartate (NMDA) receptor antagonist memantine. Donepezil can improve cognitive function in patients with mild to moderate impairment. Memantine is mainly prescribed to alleviate cognitive decline and behavioral symptoms in moderate to severe stages. In addition, drugs such as oxiracetam are used as adjuvant therapies to enhance cerebral metabolism. For comorbid symptoms, including depression and hallucinations, antidepressants such as sertraline and low-dose atypical antipsychotics such as risperidone are sometimes employed [4]. Nevertheless, existing drugs suffer from significant limitations. They provide only transient symptomatic relief and do not halt underlying pathological processes, such as neuronal loss and β-amyloid deposition. Their therapeutic efficacy is modest and shows marked interindividual variability, leaving some patients with minimal benefit. Single-target mechanisms are insufficient to address the multifactorial pathophysiology of cognitive impairment, particularly in Alzheimer’s disease. These drugs are also frequently associated with adverse effects, including gastrointestinal disturbances and cardiovascular risks [5]. Furthermore, most approved drugs are indicated exclusively for Alzheimer’s disease. There is limited evidence supporting their use in other subtypes, such as vascular cognitive impairment [6].

These limitations underscore the urgent need for novel therapeutic strategies in the management of cognitive impairment. Specifically, there is a need for interventions that can overcome single-target restrictions, offer favorable safety profiles, and demonstrate broad applicability. In this context, plant-derived polysaccharides (PDPs) have garnered growing attention. They exhibit multi-target mechanisms, a good safety record, and a long history of use in traditional medicine, including Traditional Chinese Medicine [7]. Recent studies have shown that PDPs possess clear neuroprotective effects through multiple pathways. These include anti-inflammatory, antioxidant, anti-apoptotic, and immunomodulatory mechanisms [8,9]. Importantly, PDPs may also provide indirect neuroprotection by modulating the “gut–brain axis.” The critical role of the gut–brain axis in the pathogenesis and progression of neurodegenerative disorders is increasingly recognized [10]. Therefore, this review aims to systematically summarize current evidence regarding the molecular mechanisms and clinical progress of PDPs in the management of cognitive impairment. Particular emphasis is placed on their translational potential, with the goal of providing valuable insights to guide future research.

## 2. Materials and Methods

### 2.1. Literature Search Strategy

This review was conducted using a structured literature search strategy to systematically identify and summarize studies investigating the neuroprotective effects of PDPs in the context of cognitive disorders. Literature searches were performed in major electronic databases, including PubMed, Web of Science, and Scopus, covering publications from the past 10 years available up to 1 October 2024.

The search strategy employed combinations of the following keywords: “plant-derived polysaccharides,” “polysaccharides,” “cognitive disorders,” “Alzheimer’s disease,” “neuroprotection,” “gut–brain axis,” “gut microbiota,” “short-chain fatty acids,” “oxidative stress,” and “neuroinflammation.” In addition, the reference lists of all eligible articles were manually screened to identify further relevant studies.

### 2.2. Inclusion and Exclusion Criteria

Studies were included if they met one or more of the following criteria: (1) in vitro or in vivo experimental studies investigating the effects of PDPs on cognitive function or related neurobiological mechanisms; (2) experimental studies examining the interactions between PDPs, gut microbiota, and the gut–brain axis; and (3) clinical studies or clinical reports evaluating the effects of PDPs on cognitive outcomes.

Studies were excluded if they met any of the following criteria: (1) review articles, conference abstracts, editorials, commentaries, or opinion papers lacking original experimental or clinical data; (2) studies focusing on non-plant-derived polysaccharides (e.g., polysaccharides of animal or microbial origin); (3) studies that did not assess cognitive function, neurological outcomes, or clearly defined neuroprotective mechanisms; (4) studies with insufficient methodological detail or incomplete data that precluded meaningful interpretation.

### 2.3. Data Extraction and Synthesis

**Figure 1:**
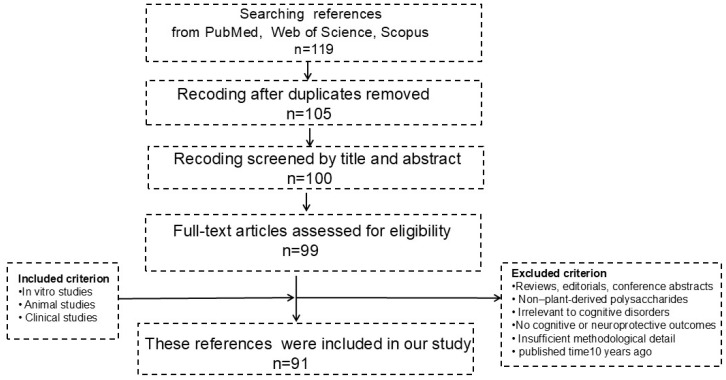
Literature search workflow.

Eligible studies were categorized according to experimental type (in vitro studies, animal studies, or clinical studies) and qualitatively synthesized based on their primary mechanisms of action, including anti-inflammatory effects, antioxidant activity, modulation of neuronal apoptosis, inhibition of protein aggregation, and regulation of gut microbiota and microbial metabolites. Given the substantial heterogeneity in polysaccharide sources, structural characteristics, and experimental designs, a qualitative synthesis approach was adopted, and no quantitative meta-analysis was performed (Figure 1).

## 3. Overview of Plant-Derived Polysaccharides

### 3.1. The Structure of Plant-Derived Polysaccharides

Plant-derived polysaccharides (PDPs) are high-molecular-weight biopolymers composed of monosaccharide units linked through glycosidic bonds. These complex carbohydrates are typically classified based on their monosaccharide composition (e.g., glucose, arabinose, galactose, mannose, rhamnose), branching patterns, molecular weight, and solubility [11]. Structurally, PDPs can be linear or branched. They exhibit various degrees of polymerization, which influence their physicochemical and biological properties [12]. Functionally, PDPs serve multiple roles. They can act as structural components, such as cellulose and hemicellulose, or as storage molecules, such as starch and fructans. Importantly, some PDPs function as bioactive constituents with immunomodulatory, antioxidant, and anti-inflammatory activities [13]. It is this latter category that has attracted interest for therapeutic applications, particularly for PDPs extracted from medicinal plants used in Traditional Chinese Medicine (TCM) and other ethnomedical traditions.

### 3.2. The Function of Plant-Derived Polysaccharides

A wide variety of medicinal plants are rich in bioactive polysaccharides. Prominent TCM preparations include Ganoderma lucidum (Lingzhi), which contains immunostimulatory and neuroprotective polysaccharides, primarily β-glucans [13]. Astragalus membranaceus (Huangqi) produces Astragalus polysaccharides (APS), which exhibit anti-inflammatory and antioxidant properties [14]. Lycium barbarum (goji berry) is rich in Lycium barbarum polysaccharides (LBPs), which have well-documented neuroprotective and cognitive-enhancing effects [15]. Many other PDPs are derived from medicinal plants, including Panax ginseng, Codonopsis pilosula, and Dendrobium officinale. These polysaccharides also hold promise as therapeutic agents with cognitive benefits [16], This highlights the wealth of research and development opportunities in the ethnopharmacology space. PDP-focused research is not confined to TCM. Polysaccharides isolated from other plants, such as Aloe vera, Allium cepa (onion), and Opuntia ficus-indica (prickly pear), also show potential modulatory effects in neurodegeneration [17,18,19]. Plant polysaccharides possess a variety of pharmacological activities. They can enhance immunity, provide neuroprotection, regulate blood glucose, exert antitumor effects, and exhibit antioxidant properties.

### 3.3. The Relationship of Structure and Function of Plant-Derived Polysaccharides

**Figure 2:**
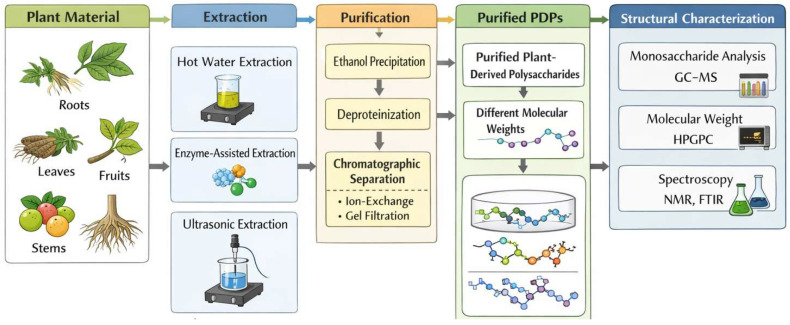
The method of extraction and purification of plant polysaccharides.

The biological activity of PDPs is closely linked to their structural features, which are influenced by the extraction and purification methods employed [20,21]. Common extraction techniques include hot water extraction, enzyme-assisted extraction, and ultrasonic or microwave-assisted extraction. Following extraction, purification often involves ethanol precipitation, deproteinization, and chromatographic separation (e.g., ion-exchange or gel-filtration chromatography) [21]. After isolation, the structural and compositional characterization of these polysaccharides can be performed using various analytical techniques. These techniques include monosaccharide composition analysis (e.g., gas chromatography-mass spectrometry, GC-MS), molecular weight determination (e.g., high-performance gel permeation chromatography), and spectroscopic methods (e.g., NMR, FTIR) [21]. However, a major challenge in the field is the lack of standardized protocols, which complicates cross-study comparisons and translational development [22]. Differences in plant source, environmental conditions, and processing techniques can produce polysaccharides with varying bioactivity. This variability may contribute to inconsistent findings across studies and highlights the need for systematic efforts to carefully document the effects of specific PDPs in appropriate experimental model systems (Figure 2).

## 4. Molecular Mechanisms Governing Pdp-Mediated Neuroprotection

**Figure 3:**
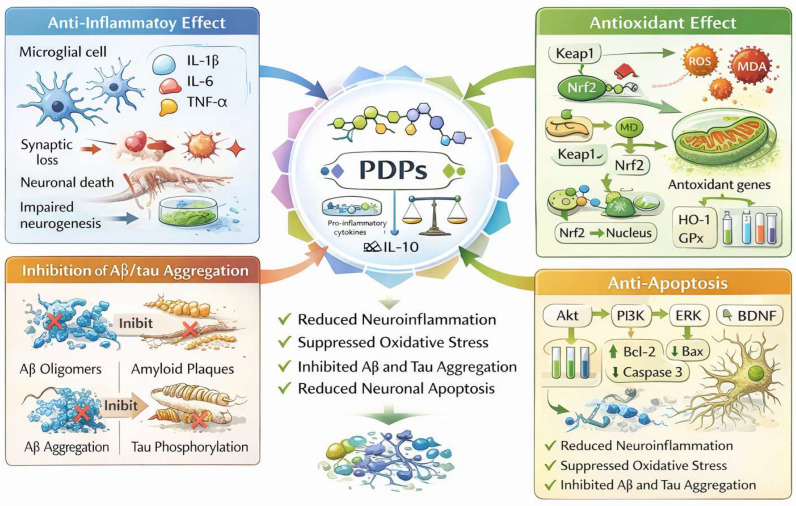
Molecular mechanisms of plant polysaccharides.

The neuroprotective potential of PDPs is attributed to their ability to interact with multiple molecular targets and biological pathways implicated in cognitive dysfunction. In this section, we summarize key mechanisms through which PDPs may exert therapeutic effects in neurodegenerative diseases (Figure 3).

### 4.1. The Anti-Inflammatory Effect

Chronic neuroinflammation is prevalent in various cognitive disorders, particularly in Alzheimer’s disease (AD) and vascular dementia (VaD), where overactivation of microglia and astrocytes leads to the release of pro-inflammatory cytokines (e.g., IL-1β, IL-6, TNF-α), resulting in synaptic loss, neuronal death, and impaired neurogenesis [23,24,25,26]. Polysaccharides (PDPs) exert anti-inflammatory effects through multiple mechanisms: they inhibit inflammatory signaling pathways such as NF-κB, MAPK, and TLR4/MyD88 to reduce pro-inflammatory cytokine levels and increase the expression of anti-inflammatory factors such as IL-10, and they modulate glial cell activation to maintain central nervous system homeostasis [27]. Studies have shown that Astragalus polysaccharides improve learning and memory deficits in AD model rats by suppressing NF-κB signaling [28]; Poria cocos polysaccharides reduce IL-1β and TNF-α levels in the hippocampus, alleviating cognitive decline [29]. Dendrobium officinale polysaccharides provide neuroprotection by regulating microglial activation and downregulating IL-1β, TNF-α, IL-6, and IL-10, improving AD-related cognitive deficits [30]. Houshan Dendrobium polysaccharides ameliorate D-galactose-induced cognitive impairment in mice by reducing inflammation [31]. In addition, evaluations using the Y-maze, passive avoidance test (PAT), and Morris water maze (MWM) demonstrated that Lycium barbarum polysaccharides not only decreased NF-κB, TNF-α, IL-1β, and IL-6 levels but also increased IL-4 and IL-10 activity in the cortex, showing significant inhibitory effects on AD phenotypes [32]. In summary, PDPs exhibit prominent neuroprotective and cognitive benefits by regulating inflammatory signaling pathways, modulating glial cell activation, and balancing pro- and anti-inflammatory factors.

### 4.2. The Antioxidant Effect

Oxidative stress, resulting from the overproduction of reactive oxygen species (ROS) and impaired antioxidant defense, is closely linked to neuronal injury and cognitive decline [33]. PDPs exhibit strong free radical scavenging activity and upregulate endogenous antioxidant enzymes such as superoxide dismutase (SOD), catalase, and glutathione peroxidase (GPx) [34]. For example, LBP treatment can attenuate lipid peroxidation and ROS accumulation in hippocampal neurons, thereby preserving mitochondrial integrity and synaptic function [35]. Polysaccharides from *Hyriopsis cumingii* improved SOD activity, reduced lipid peroxidation (MDA), and lowered caspase-3 expression in brain tissue, suggesting neuroprotective effects [36]. Polysaccharides from *Gynostemma pentaphyllum* (GPP1) attenuated Aβ-induced ROS accumulation, lipid peroxidation, and apoptotic markers in PC12 cells. Other examples include antioxidant and cytoprotective activities from *Ganoderma lucidum*, *Saccharina japonica*, and *Angelica sinensis* polysaccharides, which prevented ROS accumulation, preserved mitochondrial function, and reduced apoptosis in cell and animal models [37,38,39,40]. *Cynomorium songaricum* polysaccharides also conferred resistance to oxidative injury in neuronal cultures [41]. At the molecular level, many of these effects are linked to activation of the Nrf2/HO-1 signaling pathway, which serves as a major regulator of cellular antioxidant responses. Nrf2 (nuclear factor erythroid 2–related factor 2) is a transcription factor that senses oxidative stress and orchestrates the expression of a broad array of cytoprotective genes. Under basal conditions, Nrf2 is sequestered in the cytoplasm by Keap1 (Kelch-like ECH-associated protein 1) and targeted for ubiquitin-mediated degradation. Upon oxidative or electrophilic stress, Nrf2 dissociates from Keap1, translocates into the nucleus, and binds to antioxidant response elements (AREs) in the promoter regions of target genes, including glutathione-related enzymes, HO-1, NADPH quinone oxidoreductase 1, and thioredoxin systems [42]. Nrf2 controls the transcription of various cytoprotective enzymes through the Keap1/Nrf2/ARE axis [43], including glutathione-related enzymes, HO-1, NADPH quinone oxidoreductase 1, and thioredoxin systems. LBPs, for example, have been shown to enhance Nrf2 and HO-1 expression in rodent retinal tissue following ischemic injury, implicating this pathway in its neuroprotective action. By restoring redox balance, PDPs help maintain cellular homeostasis and protect against age-related oxidative damage in the brain, providing an avenue to better cognitive outcomes [44,45].

### 4.3. Inhibition of Protein Aggregation

Abnormal protein deposition is a key pathological feature of AD and other dementias. For example, Aβ accumulation in the brain and hyperphosphorylation of tau protein can lead to synaptic damage and neuronal loss [30]. Some polysaccharides (PDPs) have been shown to: inhibit excessive Aβ production, promote Aβ clearance, and reduce abnormal tau phosphorylation [44,45]. For instance, Lycium barbarum polysaccharides have been found to decrease Aβ levels and improve cognitive performance in AD mouse models [46]. Hericium erinaceus polysaccharides can ameliorate tau protein deposition, alleviating AD pathology [46]. Schisandra chinensis polysaccharides can regulate metabolites in the hippocampus to inhibit tau protein phosphorylation, reduce acetylcholinesterase activity, and improve Alzheimer’s disease-related deficits [47].

### 4.4. Inhibit Neuronal Cell Apoptosis

Apoptotic cell death of neurons is a critical contributor to cognitive dysfunction. PDPs can modulate the expression of key regulators of apoptosis, including Bax, Bcl-2, and caspase-3, shifting the balance toward cell survival. Simultaneously, many PDPs activate pro-survival signaling cascades, such as the PI3K/Akt and MAPK/ERK pathways [48]. These pathways are also involved in regulating the expression of brain-derived neurotrophic factor (BDNF), a crucial molecule for synaptic plasticity, learning, and memory [49]. Enhanced BDNF signaling has been observed in animal models treated with PDPs, suggesting their role in promoting neuronal resilience and regeneration [50,51]. These regulatory mechanisms are also often complementary. For example, Cactus-derived polysaccharides can limit ROS accumulation and prevent apoptosis in H_2_O_2_-treated PC12 cells, normalizing the Bax/Bcl-2 ratio [52].

While these examples offer a glimpse into the capacity of PDPs for neuroprotection at a molecular level, further work is needed to fully characterize the dynamics of these activities across cognitive disorders and in clinically relevant model systems, informing more targeted interventional efforts.

## 5. Pdps as Regulators of the Gut–Brain Axis

An emerging area of interest is the influence of PDPs on the gut–brain axis, the bidirectional communication network between the gastrointestinal tract and the central nervous system. Dysbiosis of the gut microbiota has been implicated in neuroinflammation, amyloid deposition, and cognitive impairment.

The biological effects of polysaccharides are largely dependent on their microbiota-mediated metabolism in the host. Because humans lack endogenous enzymes capable of degrading complex polysaccharides, planter natural product derived polysaccharides (PDPs/NPPs) are primarily fermented by the gut microbiota in the colon. Their structural characteristics including monosaccharide composition, glycosidic linkage types, degree of branching, and molecular weight critically determine fermentability, microbial selectivity, and the resulting metabolite profiles. Distinct polysaccharide structures can therefore selectively enrich specific functional microbial taxa, such as Bifidobacterium, Lactobacillus, Faecalibacterium prausnitzii, or *Roseburia* spp., thereby driving differential production of short-chain fatty acids (SCFAs) and other bioactive metabolites [53]. Plant polysaccharides must reach the colon to be metabolized by the gut micro-biota, which express carbohydrate-active enzymes (CAZymes) that break them down into bioactive metabolites [54,55].

SCFAs generated in response to PDP/NPP fermentation represent a central mechanistic link connecting polysaccharide structure, microbial ecology, and host physiological outcomes. Acetate, propionate, and butyrate not only serve as major energy substrates for intestinal epithelial cells and support oxidative phosphorylation but also activate G protein-coupled receptors such as GPR41 and GPR43, stimulating the secretion of gut hormones including glucagon-like peptide-1 (GLP-1) and peptide YY (PYY). Through these pathways, SCFAs regulate appetite, insulin sensitivity, and systemic glucose and lipid homeostasis. In parallel, SCFAs exert immunomodulatory effects by inhibiting histone deacetylase (HDAC) activity, promoting regulatory T cell (Treg) differentiation, suppressing pro-inflammatory cytokine production, and upregulating tight junction proteins, thereby preserving intestinal barrier integrity and limiting chronic low-grade inflammation [54,55].

Along the gut–brain axis, structurally diverse polysaccharides selectively enrich microbial populations involved in γ-aminobutyric acid (GABA) and tryptophan metabolism, leading to changes in SCFAs, GABA, and indole-derived metabolites. These microbial products influence vagal signaling, blood–brain barrier integrity, neuroinflammatory status, and neurotrophic factor expression (e.g., BDNF), thereby shaping metabolic and neuroendocrine outcomes. Collectively, these findings highlight that PDPs/NPPs exert systemic effects not through a single pathway, but via a structure-dependent fermentation process that generates distinct metabolite signals and coordinates multi-organ crosstalk [56,57].

One common marker of microbial imbalance is the Firmicutes/Bacteroidetes (F/B) ratio, which is often elevated in conditions like obesity, inflammation, and neuropsychiatric disorders. Various polysaccharides, including those from *Polygonatum sibiricum*, *Panax ginseng*, *Astragalus membranaceus*, and *Dendrobium officinale* [58,59,60,61], have been shown in animal studies to normalize the F/B ratio and improve behavioral symptoms. Interestingly, some polysaccharides from *Lycium barbarum* and *Abelmoschus esculentus* improved cognitive and emotional outcomes even while increasing the F/B ratio [62,63,64]. These findings suggest that the absolute F/B ratio may not be the primary determinant of host health. Instead, the functional composition of Firmicutes—particularly the enrichment of short-chain fatty acid–producing bacteria such as Roseburia and Faecalibacterium—appears to play a more critical role in mediating beneficial effects.

Polysaccharides from *Astragalus*, *Hippophae rhamnoides*, and *Prunus persica* have been reported to enhance the abundance of these beneficial bacteria and ameliorate cognitive or mood-related deficits in mice [8,65]. At the genus level, increases in *Lactobacillus* and *Bacteroides* species are frequently observed after polysaccharide treatment and are associated with neuroprotective effects via anti-inflammatory and antioxidant mechanisms. Other genera such as *Coprococcus*, *Prevotella*, and *Paraprevotella* have also been implicated in mediating the neuroprotective effects of polysaccharides through roles in neurotransmitter metabolism and immune regulation [56,66]. For example, Transplantation of *Coprococcus eutactus* alleviated depressive-like behaviors induced by chronic restraint stress in mice, prevented synaptic loss and glial inflammation, and shifted tryptophan metabolism from the neurotoxic kynurenine pathway toward serotonin production in both the brain and colon. Mechanistically, *Coprococcus eutactus* inhibited rate-limiting enzymes in the kynurenine pathway, while restoring colonic goblet cells and mucus secretion. These findings reveal the antidepressant potential of *Coprococcus eutactus* and its role in linking gut microbiota modulation to neurotransmitter balance [56,66].

Most plant polysaccharides reach the large intestine undigested and are fermented by gut microbes into various metabolites, with SCFAs such as acetate, propionate, and butyrate being among the most prominent [67,68]. These short-chain fatty acids (SCFAs) play vital roles in maintaining gut health, modulating inflammation, supporting the intestinal barrier, and influencing brain function via the gut–brain axis. SCFAs act through several mechanisms, including activation of G-protein-coupled receptors, inhibition of histone deacetylases, and serving as energy substrates [26]. These actions contribute to reduced neuroinflammation, improved cognition, and stabilization of mood in animal models. In most studies published to date, plant polysaccharides increased SCFA levels in the gut, particularly butyrate, which is known for its neuroprotective and anti-inflammatory properties [56,69,70]. Polysaccharides from the leaves of Polygonatum sibiricum (PsPs) were extracted, purified, and characterized, showing a total sugar content of 97.48% and a monosaccharide composition of mannose, rhamnose, galacturonic acid, glucose, xylose, and arabinose at a molar ratio of 6.6:15.4:4.5:8.8:40.7:24. In mice, PsPs treatment modulated the intestinal microbiota, increasing the relative abundance of Firmicutes and Lactobacillus while decreasing Bacteroidetes, Lachnospiraceae, and Bacteroides. Importantly, PsPs significantly increased the levels of short-chain fatty acids (SCFAs), with acetic acid, propionic acid, and butyric acid all elevated. Notably, butyric acid content nearly doubled, rising from 7.3 μg/mL in control mice to 15.2 μg/mL after PsPs treatment. The production of individual SCFAs is influenced by gut microbiota composition. For instance, *Bacteroides* and *Prevotella* primarily produce acetate and propionate [71], while *Faecalibacterium prausnitzii* and *Roseburia* are major butyrate producers [72]. Beyond SCFAs, other microbial metabolites such as valeric acid, lipid-like molecules, and unsaturated fatty acids have been associated with improved cognitive function after polysaccharide treatment [8,73]. Ganoderma lucidum polysaccharides can improve learning deficits in D-galactose-induced aging mice by altering the distribution of Lactobacillus in the gut and regulating peptidoglycan and secondary bile acid synthesis [74]. Barley polysaccharides can ameliorate mild cognitive impairment in mice via the liver–brain–gut axis. This involves increasing the abundance of gut microbes norank_f_Muribaculaceae and unclassified f_Lachnospiraceae, as well as elevating SCFA levels, particularly butyrate [75]. *Cistanche deserticola* polysaccharides can improve cognitive function in D-galactose-induced aging mice by restoring gut homeostasis [76]. In some cases, metabolomic shifts included reductions in compounds typically associated with neuropsychiatric medications, suggesting a rebalancing effect on the host metabolome.

Given the growing recognition of the gut–brain axis as a regulator of the pathogenesis of AD, VaD, and other cognitive disorders [77,78], research focused on the targeted modulation of the gut microbiota to achieve beneficial effects through the application of PDPs has clear value as a direction for further study. However, most existing studies are limited by small sample sizes, short intervention durations, heterogeneous outcome measures, and the inconsistent composition of PDP preparations.

## 6. Clinical Prospects

Human clinical evidence for the potential benefits of plant-derived polysaccharides (PDPs) has begun to emerge (Table 1). For example, in individuals with subjective cognitive impairment (SCI), Tremella fuciformis-derived polysaccharides were evaluated in an 8-week randomized, double-blind, placebo-controlled trial, in which supplementation was associated with improvements in subjective memory complaints and selected domains of short-term memory and executive function, without significant differences in adverse event rates compared with placebo [79]. Exploratory neuroimaging analyses further suggested region-specific gray matter volume changes in cognition-related brain areas. Despite these encouraging findings, the study was limited by a relatively small sample size, short intervention duration, and the exploratory nature of some outcome measures. In addition, Lycium barbarum polysaccharide (LBP), the major bioactive component of Lycium barbarum, has been evaluated as a gut microbiota-targeted intervention in patients with non-alcoholic fatty liver disease (NAFLD) in a randomized, double-blind, placebo-controlled trial. Fifty NAFLD patients diagnosed by abdominal ultrasonography, laboratory assessments, and questionnaires were randomly assigned to receive either LBP or maltodextrin placebo capsules for 3 months. The primary outcome was improvement in serum alanine aminotransferase (ALT) levels, while secondary outcomes included changes in gut microbiota composition and diversity, intestinal permeability, body composition, treatment compliance, and adverse events. This study provides preliminary evidence supporting the feasibility and short-term safety of LBP supplementation for modulating gut microbiota in NAFLD patients [80,81].

Overall, these findings suggest that PDPs may have potential clinical benefits in both cognitive and metabolic disorders, but the limited sample sizes, short intervention durations, and heterogeneity of outcome measures highlight the need for further well-powered, long-term randomized controlled trials to confirm efficacy and elucidate underlying mechanisms. Moreover, clinical translation must consider regulatory pathways for botanical products, ethical review for human trials, and practical considerations such as patient adherence, formulation stability, and multicenter coordination. Establishing consensus on standardized outcome measures and quality control parameters will facilitate regulatory evaluation and broader clinical adoption.

LBPs are among the most studied PDPs in clinical settings, as evidenced by their robust anti-inflammatory and antidepressive activity in patients affected by nonalcoholic fatty liver disease and subthreshold depression [80,81]. LBP preparations have also exhibited superior efficacy in preclinical models of AD and cognitive decline, but no published trials have specifically tested LBP samples in the management of AD, VaD, or other prominent cognitive disorders. Likewise, while *Ganoderma lucidum* polysaccharides have been well-validated as safe, hepatoprotective options in healthy volunteers [82], the clinical evidence regarding their performance has been limited. *Cordyceps sinensis* and *Ganoderma lucidum* reportedly failed to improve the cognitive function of healthy subjects [83]. The lack of cognitive improvement observed with O. sinensis and G. lucidum supplementation in young healthy participants may be due to the short 30-day intervention, the ceiling effect in cognitively normal individuals, limited sample size, and uncertain bioavailability of active compounds. Additionally, cognitive assessment tools may have been insufficiently sensitive to detect subtle changes in this population. These factors likely contributed to the absence of measurable benefits. And a similarly disappointing results were noted in a study of *Ganoderma lucidum* spore powder in the context of AD such that patients failed to exhibit improvements in key indices (NPI, ADAS-cog) [84]. The lack of significant effects of *Ganoderma lucidum* in this pilot study may be due to the short 6-week treatment duration, small sample size, the preliminary design focused on feasibility and safety, and individual variability in disease progression, all of which could limit the detection of cognitive improvements in ADAS-cog, WHOQOL-BREF, and NPI scores. Given the inconsistent formulation of these preparations, limited intervention duration, and variable outcome measures, future research should prioritize the development of standardized extracts, rigorous dosing studies, and longitudinal assessment to determine both efficacy and safety.

*Astragalus membranaceus* polysaccharides (APS) have been included in several compound formulations evaluated in Chinese clinical studies for memory enhancement and aging-related cognitive decline. For instance, a double-blind, randomized, controlled preliminary study enrolling 61 participants demonstrated the ability of these polysaccharides to mitigate several cognitive symptoms associated with post-stroke fatigue [85]. Clinical trials have been initiated to explore the utility of APS in AD [86], but the results of these trials have yet to be published and will represent an important step toward the commercial implementation of these plant derivatives. Future studies should incorporate standardized APS preparations, longer follow-up durations, and harmonized outcome measures, while also considering regulatory guidance and ethical oversight for trials involving vulnerable populations such as elderly or cognitively impaired patients.

The limitations of this review can be summarized in several aspects. First, the number of available clinical studies is limited, and the sample sizes are generally small, with most enrolling fewer than 100 participants, resulting in limited statistical power [83]. The study durations are also relatively short, which may be insufficient to observe the long-term cognitive benefits or disease-modifying effects of PDPs. Second, the composition and extraction methods of PDP formulations vary across studies and lack standardization, affecting the reproducibility of results and posing challenges for regulatory approval. Third, many studies use compound herbal formulas with complex components, making it difficult to determine the specific contribution of individual PDPs. Finally, data on the long-term safety and pharmacovigilance of PDPs are still insufficient. Overall, these factors limit a comprehensive assessment of the clinical value of PDPs. Overall, these limitations underscore the need for a coordinated research strategy that includes standardization of PDP preparations, rigorous study design, long-term follow-up, and clear consideration of regulatory, ethical, and operational factors to support safe and effective clinical translation.

## 7. Future Perspectives and Opportunities

While the preclinical and early clinical evidence clearly points to the medicinal utility of PDPs, in line with their long history of traditional use in TCM and other practices throughout the world, a series of issues remain to be addressed to support their broader clinical uptake.

The bioavailability of orally administered PDPs is generally low due to their large molecular size and resistance to digestion (the bioavailability less than 50%) [87]. However, their interaction with gut microbiota leads to fermentation and production of bioactive metabolites such as SCFAs [88], which may mediate systemic and central effects. Still, detailed pharmacokinetic data (including absorption, distribution, metabolism, and excretion [ADME]) are sparse. More studies using labeled compounds or advanced imaging are needed to clarify the pathways by which PDPs or their metabolites exert neuroprotective effects.

Large-scale, randomized, placebo-controlled clinical trials are essential to evaluate the efficacy and safety of standardized PDPs. Trials should target well-defined populations (e.g., MCI, early AD), employ validated cognitive endpoints (e.g., MoCA, ADAS-Cog), and include long-term follow-up. Where possible, biomarker analyses should be integrated to support mechanistic understanding. Furthermore, future research should prioritize addressing key knowledge gaps in a systematic manner: standardization of polysaccharide characterization, optimization of dosage regimens, administration routes, and intervention duration, as well as evaluation of long-term safety and pharmacokinetics. This prioritization will help focus efforts on the most critical areas for clinical translation.

Adoption of modern extraction methods such as ultrasonic-assisted, enzyme-assisted, and supercritical fluid extraction can enhance yield and preserve functional moieties [89]. Structural analysis using multi-dimensional NMR, MALDI-TOF MS, and chromatographic profiling can help define pharmacophores and optimize formulations [90], offering further insights to inform ongoing research and development efforts. Additionally, regulatory, ethical, and practical considerations for clinical translation should be addressed, including ensuring product quality, compliance with guidelines, and feasibility of long-term administration in target populations.

## 8. Limitations

Despite the growing body of evidence supporting the potential neuroprotective effects of plant-derived polysaccharides, several limitations of the present review should be acknowledged. First, the majority of studies included in this review are based on in vitro experiments and animal models. Although these preclinical studies provide important mechanistic insights, their translational relevance to human cognitive disorders remains uncertain, and the observed effects may not be directly extrapolated to clinical settings.

Second, plant-derived polysaccharides exhibit substantial heterogeneity in terms of botanical origin, extraction methods, molecular weight, monosaccharide composition, and structural features. This heterogeneity complicates direct comparisons across studies and limits the generalizability of the reported findings. Differences in polysaccharide characterization and dosage regimens further contribute to variability in experimental outcomes.

Third, clinical evidence evaluating the efficacy of plant-derived polysaccharides in cognitive disorders remains limited. Most available clinical studies are characterized by small sample sizes, short intervention durations, heterogeneous outcome measures, and insufficient long-term follow-up. As a result, conclusions regarding clinical efficacy should be interpreted with caution.

In addition, the oral bioavailability and pharmacokinetic properties of plant-derived polysaccharides are not yet fully characterized, and long-term safety data are scarce. Inter-individual variability in gut microbiota composition may further influence the metabolic fate and biological activity of polysaccharides, potentially contributing to inconsistent therapeutic responses.

Finally, as a narrative review, this study did not apply a formal quantitative quality assessment or risk of bias scoring system to individual studies, which may introduce selection bias. Future research should prioritize standardized polysaccharide characterization, well-designed randomized controlled trials, and integrative approaches linking polysaccharide structure, gut microbial metabolism, and clinical outcomes.

## 9. Conclusions

PDPs represent a promising class of natural compounds with the potential to mitigate cognitive decline through diverse mechanisms, including anti-inflammatory, antioxidant, and neuroprotective activities, as well as through the modulation of the gut–brain axis. Polysaccharides from *Ganoderma lucidum*, *Astragalus membranaceus*, *Lycium barbarum,* and other traditional medicinal plants have shown efficacy in animal models, along with encouraging benefits in human trials. Despite their therapeutic promise, challenges related to standardization, bioavailability, and translational validation must be addressed. Advancing the clinical utility of PDPs will require rigorous trials, modern analytical methodologies, and integrative treatment models. As the global burden of cognitive disorders grows, particularly among aging populations, PDPs offer a novel and holistic avenue for intervention. With continued research and development, these complex natural macromolecules could become important tools in the prevention and management of neurodegenerative diseases.

## Figures and Tables

**Table 1:** Clinical Evidence for Plant-Derived Polysaccharides in Cognitive and Metabolic Disorders.

DP Source	Study Design	Population	Sample Size	Intervention/Dose	Duration	Primary Outcomes	Secondary Outcomes	Main Findings	Quality/Notes
*Tremella fuciformis*	RCT, double-blind, placebo-controlled	Individuals with subjective cognitive impairment (SCI)	75	Tremella fuciformis polysaccharides	8 weeks	Subjective memory complaints, short-term memory, executive function	Neuroimaging: region-specific gray matter volume changes	Improved subjective memory and selected cognitive domains	Small sample, short intervention, exploratory outcomes [79]
*Lycium barbarum* (LBP)	RCT, double-blind, placebo-controlled	NAFLD patients	50	LBP or maltodextrin	3 months	Serum ALT	Gut microbiota com-position/diversity, intestinal permeability, body composition, compliance, adverse events	Feasible and safe; modulated gut micro-biota	Small sample, short-term intervention [80]
*Lycium barbarum*	Multi-center/composite outcomes	Subthreshold depression, NAFLD	29	LBP preparations	6 weeks	Inflammatory markers, depression scores	-	Demonstrated anti-inflammatory and antidepressant activity	Preliminary data, limited evidence [81]
*Ganoderma lucidum*	RCT, safety assessment	Healthy volunteers	42	Polysaccharide preparation	6 months	Liver function	Safety assessment	Safe and hepato-protective	Short intervention, no cognitive benefit [82]
*Cordyceps sinensis* & *Ganoderma lucidum*	RCT	Healthy young adults	96	Polysaccharide preparation	30 days	Cognitive function	-	No improvement in cognition	Short duration, small sample, possible ceiling effect [83]
*Ganoderma lucidum* spore powder	Pilot study	AD patients	42	Spore powder	6 weeks	ADAS-cog, NPI, WHOQOL-BREF	-	No significant cognitive improvement	Small sample, short intervention, feasibility study [84]
*Astragalus membranaceus* (APS)	RCT, double-blind, con-trolled	Post-stroke fatigue patients	61	APS-containing compound formulation	28 days	Cognitive symptoms	-	Alleviated several cognitive symptoms	Preliminary study [85]
APS-containing formulations	Clinical trials	AD patients	66	APS-containing compound	24 weeks	Cognitive indices	-	Results not yet published	Study on-going [86]

## Data Availability

No new data were created or analyzed in this study. Data sharing is not applicable to this article.
